# Point of Care Ultrasonographic Life Support in Emergency (PULSE)—a quasi-experimental study

**DOI:** 10.1186/s12245-023-00525-w

**Published:** 2023-08-09

**Authors:** Noman Ali, Abdul Ahad Chhotani, Sannia Perwaiz Iqbal, Salman Muhammad Soomar, Ahmed Raheem, Shahan Waheed

**Affiliations:** 1 Department of Emergency Medicine, Aga Khan University, Karachi, Pakistan; 2 Department of Family Medicine, Bahria University of Health Sciences, Karachi, Pakistan

**Keywords:** Point of care, Ultrasound, Life support, Emergency

## Abstract

**Background:**

Many physicians use point-of-care ultrasound (PoCUS) in their clinical practice to improve their diagnostic capabilities, accuracy, and timeliness. Over the last two decades, the use of PoCUS in the emergency room has dramatically increased. This study aimed to determine emergency physicians’ retention of knowledge and skills after a brief training workshop on a focused ultrasound-guided approach to a patient presenting with undifferentiated shock, shortness of breath, and cardiac arrest in the emergency department of a tertiary care hospital. The secondary aim was to deliver the PoCUS-guided algorithmic approach to manage a patient presenting with undifferentiated shock, respiratory distress, and cardiac arrest in the emergency department.

**Methods:**

A quasi-experimental study was conducted with a single-day Point of Care Ultrasonographic Life Support in Emergency (PULSE) training workshop in October 2021 at the Aga Khan University Hospital, Karachi, Pakistan. A total of 32 participants attended the course, including twenty-one junior residents (PGY 1 and 2) and medical officers with experience of fewer than two years working in different emergency departments of urban tertiary care hospitals across Karachi, Pakistan. Pre- and post-assessment tools comprised a written examination, evaluating participants' knowledge and skills in ultrasound image acquisition and interpretation. Cronbach's alpha was used to calculate the validity of the tool. Results obtained before and after the training session were compared by the McNemar’s test. A *p* value of ≤ 0.05 was considered significant.

**Results:**

There was a significant improvement in response to each question pre to post-test after completion of the course (Table 1). The significant change can be seen in questions 7, 8, 13, and 15, with a percentage change of 33.3, 80.9, 42.9, and 47.7. There was a significant improvement in the understanding and knowledge of participants after the training. The scores in the post-test were high compared to the pre-test in each category, i.e., respiratory distress (*p* < 0.017), cardiac arrest (*p* < 0.041), basic ultrasound knowledge (*p* < 0.001), and undifferentiated shock (*p* < 0.001).

**Conclusion:**

All participants showed improvement in their knowledge and confidence regarding using PoCUS in life-threatening conditions. Through this study, we have also developed an algorithmic approach to managing undifferentiated shock, respiratory failure, and cardiac arrest. Future studies must assess the effectiveness and feasibility of incorporating these algorithms into clinical practice.

**Supplementary Information:**

The online version contains supplementary material available at 10.1186/s12245-023-00525-w.

## Introduction

Point of Care Ultrasound (PoCUS) refers to focused ultrasonography performed by a clinician at the patient’s bedside to acquire, interpret and integrate the findings into the immediate care of a patient [1]. In contrast to the traditional ultrasound examinations that involve providers other than the treating physicians, PoCUS examinations involve the same physician determining the need for a focused examination and can be performed in both stable and unstable patients parallel to stabilization and resuscitation [2–5].

PoCUS can help narrow the differential diagnoses, shorten the time to definitive treatment, and reduce the need for expensive stat and after-hours radiology examinations. Not only is PoCUS cost-effective and time-saving, and it has high accuracy in diagnosing and treating critically ill patients presenting to the emergency department, where access to diagnostic imaging and specialists may be constrained [5].

Over the last two decades, the use of PoCUS in the emergency department (ED) has dramatically increased [6]. By using this technique, emergency physicians can improve and expedite their clinical decision-making skills and management plans and avoid delays to definitive treatment and the need for hospital admissions [7]. Previous studies have shown the effectiveness of PoCUS in identifying and managing life-threatening conditions like respiratory or circulatory failure and cardiac arrest [8–10].

Many specialties are integrating focused ultrasound education and training into their curriculum worldwide [11]. The integration of ultrasound education in the residency programs of Pakistan is also highly variable [8]. Until now, there has been no formal training in PoCUS in Pakistan, and there is a dire need to conduct workshops and develop a curriculum of PoCUS for the physicians working in the ED of our country.

In this study, we aimed to determine the emergency physicians' retention of knowledge and skills after a brief training workshop on a focused ultrasound-guided approach to a patient presenting with undifferentiated shock, shortness of breath, and cardiac arrest in the ED of a tertiary care hospital.

Our secondary aim was to deliver the point-of-care ultrasound-guided algorithmic approach to manage a patient presenting with undifferentiated shock, respiratory distress, and cardiac arrest in the ED.

## Methods

### Study design and settings

A quasi-experimental study was conducted in the ED of the Aga Khan University Hospital (AKUH), Karachi, Pakistan, in October 2021. AKUH is a 600 bedded major tertiary care hospital in Karachi. The ED of the AKUH has an emergency medicine (EM) residency program recognized by the College of Physicians and Surgeons Pakistan (CPSP) and 24/7 supervision of consultants.

### Study population

The residents of EM and medical officers (MOs) work in different urban tertiary care hospitals across Karachi, Pakistan.

### Eligibility criteria

The study enrolled junior EM residents (year I and II training) and medical officers working in different urban tertiary care hospitals across Karachi, Pakistan. Those excluded did not provide informed written consent.

### Curriculum development

The trained emergency medicine consultant of the Aga Khan University Hospital developed the PULSE curriculum. The curriculum drew inspiration from the bedside lung ultrasonography in an emergency (BLUE) protocol, [12] rapid ultrasound in shock and hypotension (RUSH) protocol [13], and Cardiac Arrest Sonographic Assessment (CASA) exam [14] and Weingart S. Emcrit blog [15]. The curriculum was modified as per the clinical experience of the team.

The algorithms were developed to target ultrasound skills deemed relevant to managing patients presenting with undifferentiated shock, respiratory failure, and cardiac arrest. The final curriculum consists of half an hour introductory session on ultrasonography basics (knobology and physics). This was followed by a (4.5 h) session on three stations comprised of undifferentiated shock, respiratory failure, and cardiac arrest (1.5 h for each station). Each station comprises half an hour of didactic teaching and a practical demonstration on simulated patients. Practical demonstration sessions consist of PoCUS of the lung, heart, and abdomen.

Before the intervention, all participants were asked to complete an online password-protected pre-test survey. The questionnaire included 20 best-choice questions assessing baseline theoretical knowledge regarding basic ultrasound usage and participants’ image interpretation skills in managing undifferentiated shock, respiratory failure, and cardiac arrest.

**Fig. 1:**
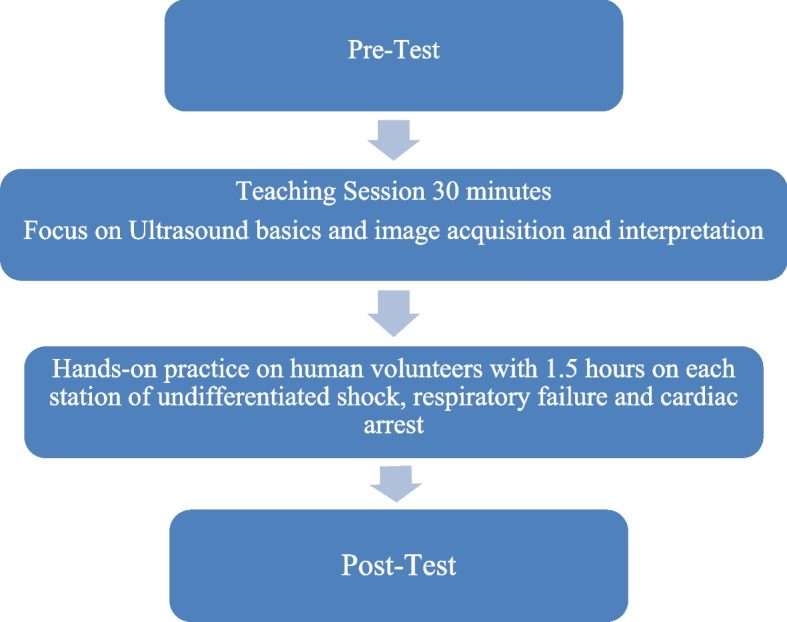
Flow diagram of the study

Upon completing the pre-test and practical component of the PoCUS workshop curriculum, all participants were asked to attempt the post-test exam (Fig. 1).

### Questionnaire

The assessment of this workshop was tested on twenty multiple-choice questions (Cronbach Alpha = 0.7%) specific to acutely ill patients, which included multiple case-based scenarios that tested specific signs, specific views, and correct image identification. Of these twenty questions, six were pertinent to respiratory distress, four were on cardiac arrest, five were undifferentiated shock, and five were on basic ultrasound knowledge.

### Outcome measures

The study’s outcome was to measure the increased knowledge and skills in image acquisition required for PoCUS-guided management of undifferentiated shock, respiratory failure, and cardiac arrest.

### Data analysis

Cronbach’s alpha was used to calculate the validity of the tool. Frequency and percentages were calculated in descriptive analysis. The results obtained before and after the training session were compared using McNemar’s test. A *p*-value ≤ 0.05 was considered statistically significant. Boxplots and line graphs were displayed to show the difference in scores with a 95% confidence interval (CI).

## Results

A total of 32 participants attended the workshop, including senior and junior residents and medical officers working in the emergency departments of different hospitals in Karachi. Out of them, twenty-one junior residents and medical officers were enrolled in the study. The study participants did not have any formal point-of-care ultrasound training. There was an improvement in response to each question pre to post-test after completion of the course (Table 1). The significant change can be seen in questions 7, 8, 13, and 15, with a percentage change of 33.3, 80.9, 42.9, and 47.7.

**Table 1:** Number of correct responses for each question pre- and post-test

Questions	Pre-test correct response *n* (%)	Post-test correct response *n* (%)	Percentage difference	*p* value
Q1	14 (66.7)	18 (85.7)	19.0	0.289
Q2	17 (81)	19 (90.5)	9.5	0.625
Q3	13 (61.9)	19 (90.5)	28.6	0.109
Q4	3 (14.3)	5 (23.8)	9.5	0.727
Q5	19 (90.5)	19 (90.5)	0.0	0.990
Q6	5 (23.8)	8 (38.1)	14.3	0.375
Q7	9 (42.9)	16 (76.2)	33.3	0.049*
Q8	3 (14.3)	20 (95.2)	80.9	< 0.001*
Q9	6 (28.6)	8 (38.1)	9.5	0.687
Q10	10 (47.6)	15 (71.4)	23.8	0.227
Q11	8 (38.1)	9 (42.9)	4.8	0.999
Q12	4 (19)	4 (19)	0.0	0.999
Q13	7 (33.3)	16 (76.2)	42.9	0.022*
Q14	15 (71.4)	19 (90.5)	19.1	0.219
Q15	4 (19)	14 (66.7)	47.7	0.006*
Q16	16 (76.2)	19 (90.5)	14.3	0.375
Q17	14 (66.7)	18 (85.7)	19.0	0.344
Q18	4 (19)	6 (28.6)	9.6	0.625
Q19	9 (42.9)	15 (71.4)	28.5	0.050
Q20	9 (42.9)	13 (61.9)	19.0	0.219

^*^significant *p*-value

**Fig. 2:**
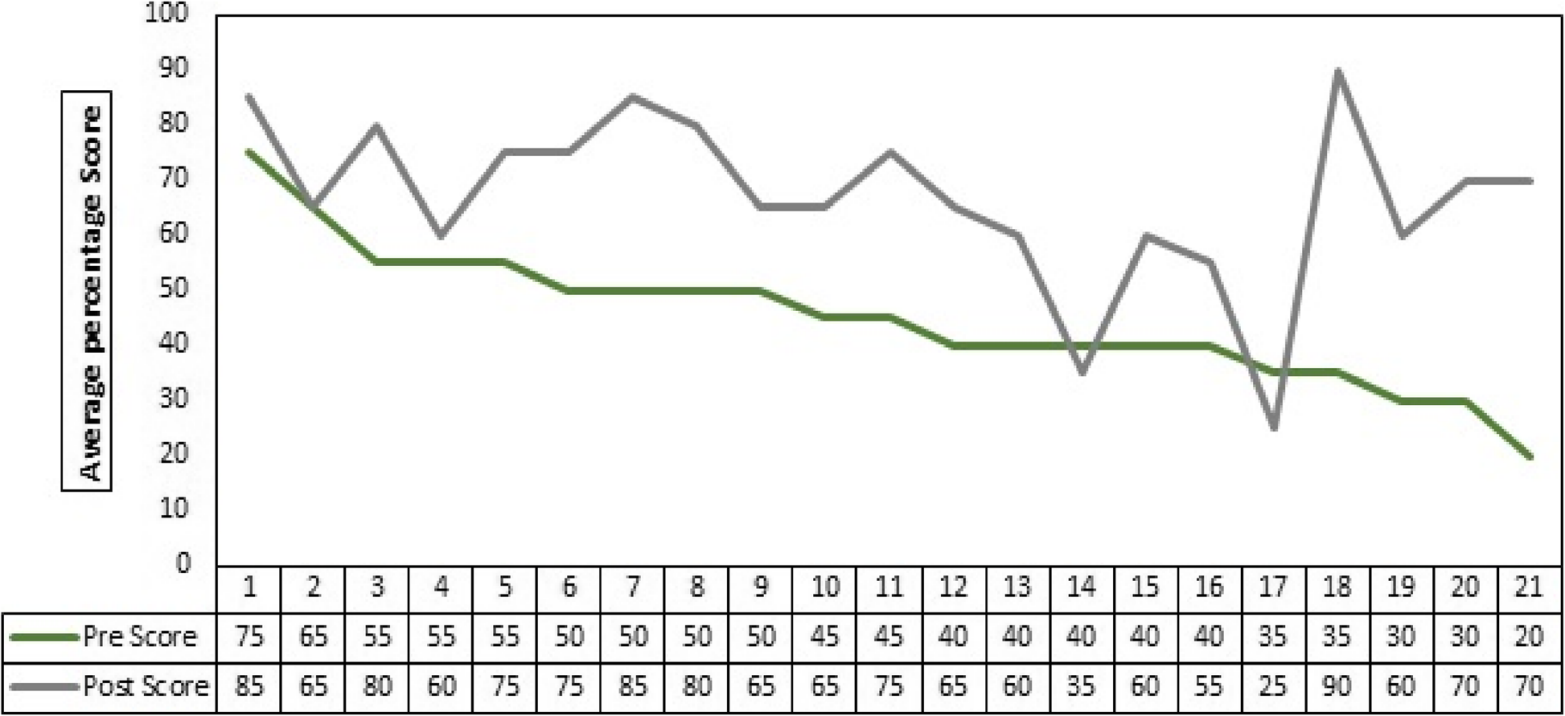
Comparison of average pre and post-test scores of individual participants

The overall post-test scores were significantly higher than the pre-test scores, and an increasing trend can be seen in Fig. 2.

**Fig. 3:**
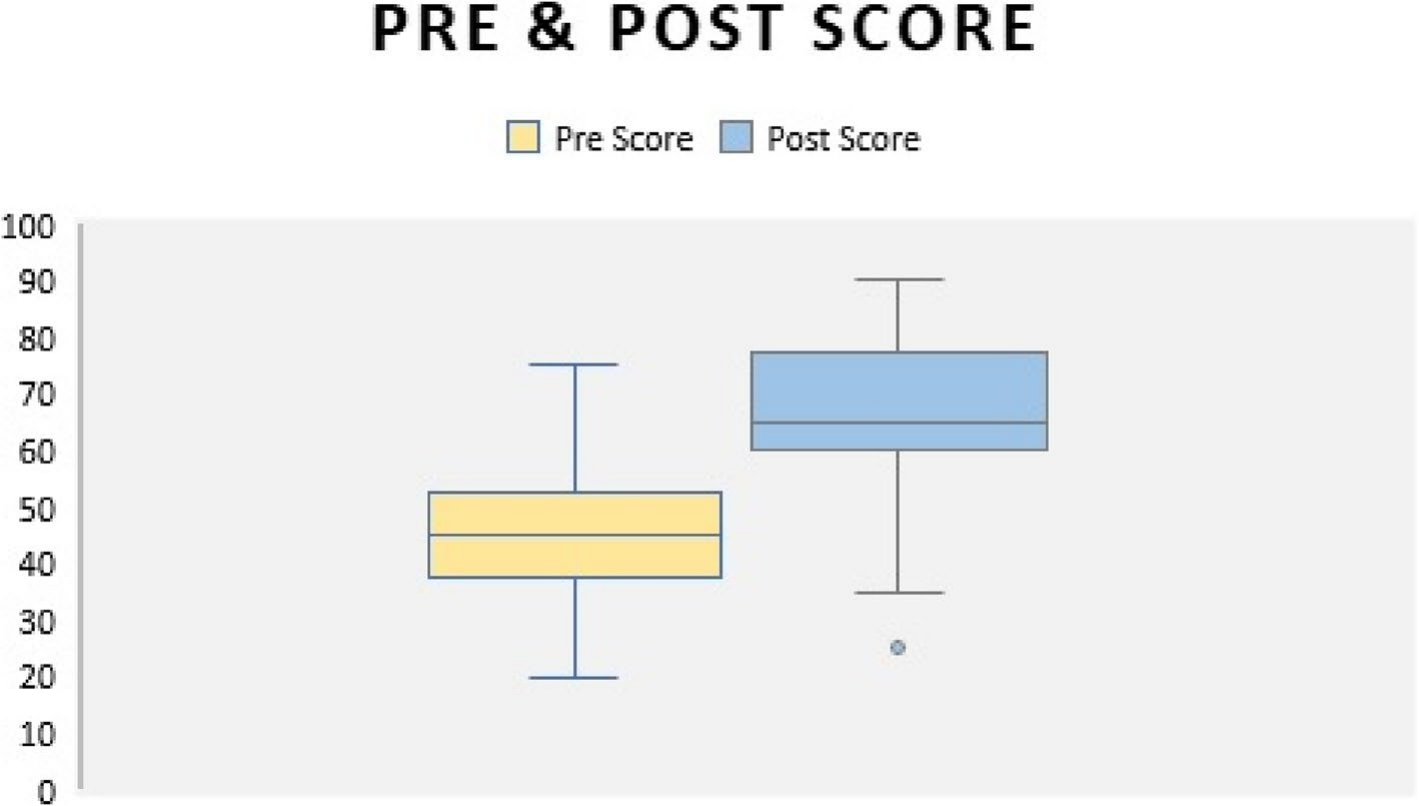
Mean score for pre and post-test for all participants

The box plot in Fig. 3 shows that the mean scores of all the study participants pre-test were 45 and the post-test 66.6, respectively.

**Fig. 4:**
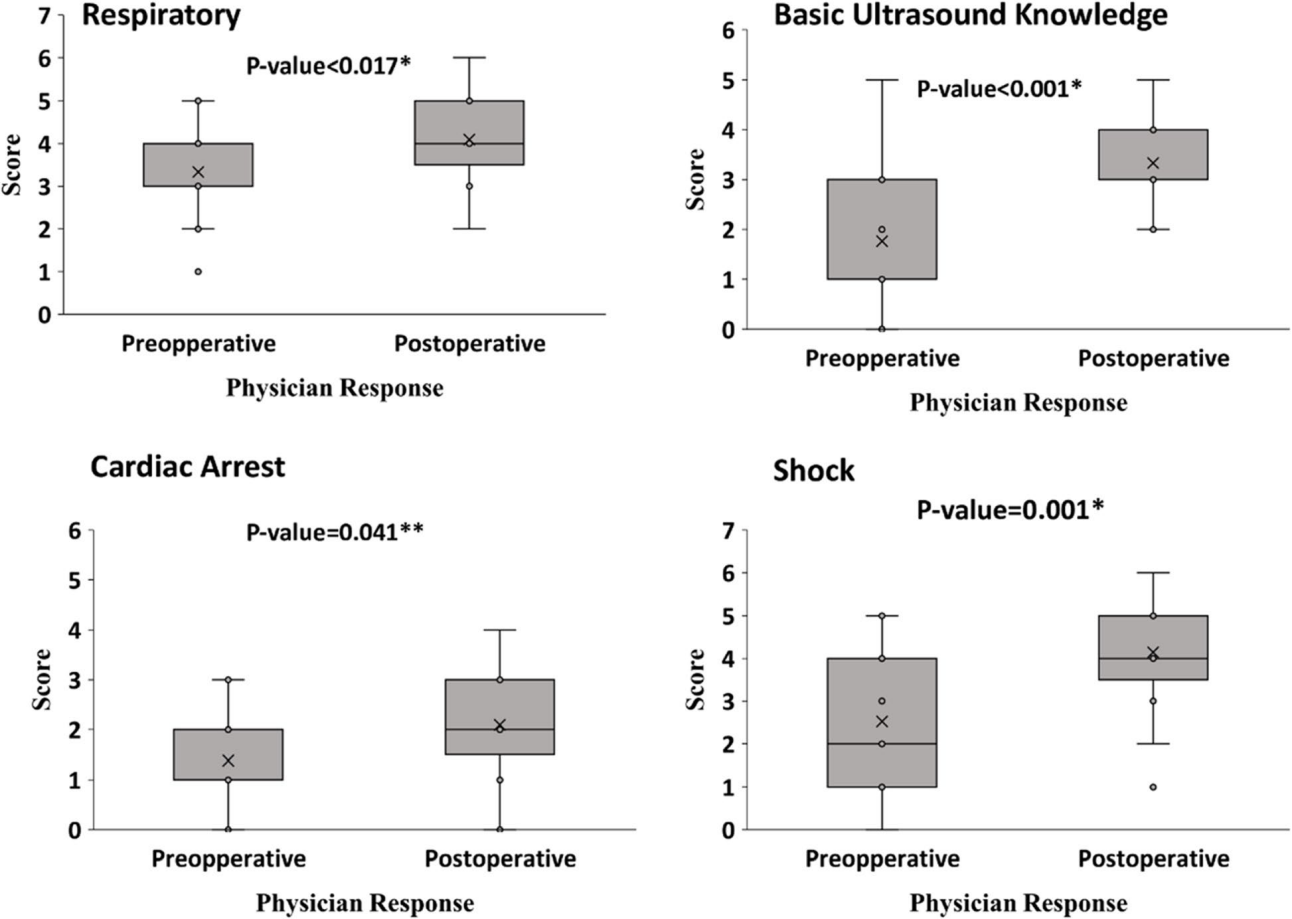
Mean score for pre and post-test for all participants stratified on categories of questions

There was a significant improvement in the understanding and knowledge of participants after the training. The scores in the post-test were high compared to the pre-test in each category, i.e., respiratory distress (*p* < 0.017), cardiac arrest (*p* < 0.041), basic ultrasound knowledge (*p* < 0.001), and undifferentiated shock (*p* < 0.001) (Fig. 4).

## Discussion

This study was conducted in a simulation-based workshop and provided promising results. Despite its small sample size, the study demonstrated improved knowledge and image interpretation skills among the participants after a brief training course. The post-workshop scores were significantly higher for the emergency medicine trainees and medical officers irrespective of the academic level, hospital practice, duration of clinical experience, and previous use of ultrasound.

The significant findings correspond to similar results of other studies [2, 16, 17]. Secondly, all the participants agreed that this would improve their clinical practice; this, too, has been reported in other similar studies [3, 4]. Finally, despite this being a short didactic simulation-based introductory course, it bore outcomes like extended ultrasound simulation workshops that were run over days [1].

All the participants reported subjective improvement in knowledge and skills acquisition after the workshop. However, a positive correlation has been observed between knowledge and skills acquisition. This can be furthered with the use of refresher courses [2, 18, 19].

This study furthered the boundary of the standardized approach by introducing a PoCUS-guided algorithmic approach to manage a patient presenting with undifferentiated shock, respiratory distress, and cardiac arrest in the emergency department (Supplementary Figure S1, S2, and S3).

Undifferentiated shock accounts for 1.3% of all emergency department presentations and is associated with high morbidity and in-hospital mortality [20, 21]. Studies have shown that the clinical examination alone is unreliable in determining the correct cause of hypotension. PoCUS has progressively become part of the standard of care in evaluating these critically ill patients [22, 23]. Through this study, we present an algorithm for assessing a patient with undifferentiated shock presented to the ED. In this algorithm, the evaluation of the patient starts with the PoCUS assessment of IVC (collapsibility index), followed by the evaluation of the heart (left ventricular dysfunction, pericardial effusion, right ventricular dilatation), lung (B-lines and lung sliding signs), and abdomen (free fluid and abdominal aortic aneurysm).

Our second algorithm is regarding the evaluation of a patient with shortness of breath. It is a common presenting complaint, with an incidence of patients coming to the ED ranging from 0.9% to 7.4% [24]. Physical examination, in addition to radiological investigation like chest x-ray, is often inaccurate in differentiating the cause of shortness of breath [22]. PoCUS is an emerging tool for evaluating a patient with shortness of breath. Many studies have shown its better performance than chest X-ray in diagnosing its etiology [24]. In this study, we have developed an algorithm for evaluating a patient who presents to the emergency department with acute shortness of breath. This algorithm allows emergency physicians to easily approach and identify the life-threatening causes of acute dyspnea.

Our third algorithm is regarding the role of PoCUS in managing cardiac arrest. PoCUS can identify the reversible causes (cardiac tamponade, tension pneumothorax, massive pulmonary embolism) and is a valuable tool in the setting of cardiac arrest [25]. Moreover, cardiac activity on ultrasound is a more reliable method of return of spontaneous circulation than a manual pulse check [26]. Pseudo-PEA is defined as a sonographic cardiac activity in the absence of a palpable pulse. The emergency physician must differentiate between the true PEA and pseudo-PEA as the latter is associated with higher odds of ROSC and survival [23].

Many clinical specialties such as emergency medicine, internal medicine, critical care, and surgery have adapted focused ultrasonography into their practice [26]. Multiple studies show that incorporating a structured, comprehensive curriculum on PoCUS in undergraduate and postgraduate training programs was instrumental in improving trainees’ knowledge and confidence in performing ultrasound-guided procedures. It significantly enhanced their interest in applying PoCUS in their future clinical practice [27–29].

Learning core point-of-care ultrasound skills can expand the emergency physicians’ clinical assessment skills, eliminating the need for extensive laboratory workup or advanced imaging. It is suggested that PoCUS should be integrated into the training of all emergency physicians and critical care physicians in Pakistan. The introduction of such longitudinal teaching programs will improve clinician knowledge and result in improved patient-centered care.

## Limitations

Our study has some limitations. Firstly, it was single-centered and with a limited sample size. The number of participants was limited due to the unavailability of trained instructors and ultrasound machines. We did not compare our single-day course with other courses due to the unavailability of data on these courses in our country, nor was our study compared to other courses of different lengths, as an optimal course length requires further evaluation.

We could not ensure long-term retention and application of the course as the post-test was conducted immediately after the workshop and only performed in a written fashion, and no follow-up study was conducted. Although the simulations were run on real people, our study lacked the use of high-fidelity simulation.

## Conclusion

After a brief training course, the study showed significant improvement in knowledge and image acquisition skills of PoCUS-guided management of critically ill patients. Through this study, we have also developed an algorithmic approach to manage undifferentiated shock, respiratory failure, and cardiac arrest. Future studies are needed to assess the effectiveness and feasibility of incorporating these algorithms into clinical practice.

### Supplementary Information

**Additional file 1: Supplementary Figure  S1–S3.** POCUS-guided algorithmic approach to manage a patient presenting with undifferentiated shock, respiratory distress, and cardiac arrest in the emergency department. 

## Data Availability

The data will be available at the reasonable request to the corresponding author at noman.ali@aku.edu.

## Abbreviations

PULSE: Point of Care Ultrasonographic Life Support in Emergency
PoCUS: Point of Care Ultrasound
CPSP: College of Physicians and Surgeons Pakistan
EM: Emergency Medicine
AKUH: Aga Khan University Hospital
