# Supplementary figures and images for: Point of Care Ultrasonographic Life Support in Emergency (PULSE)—a quasi-experimental study
Source: Int J Emerg Med. 2023 Aug 9;16:49. doi: 10.1186/s12245-023-00525-w (PMC10410962; doi:10.1186/s12245-023-00525-w)

.
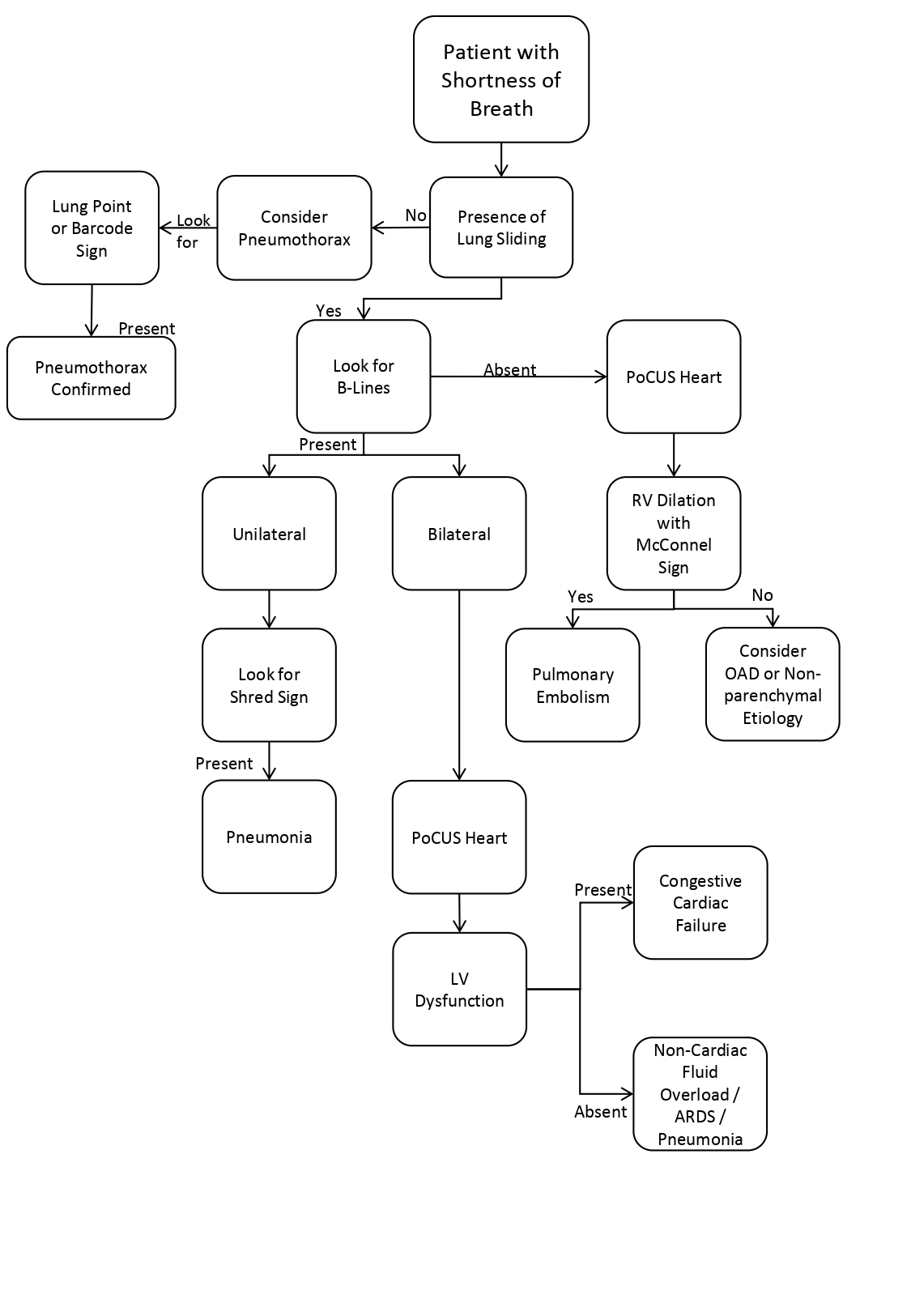


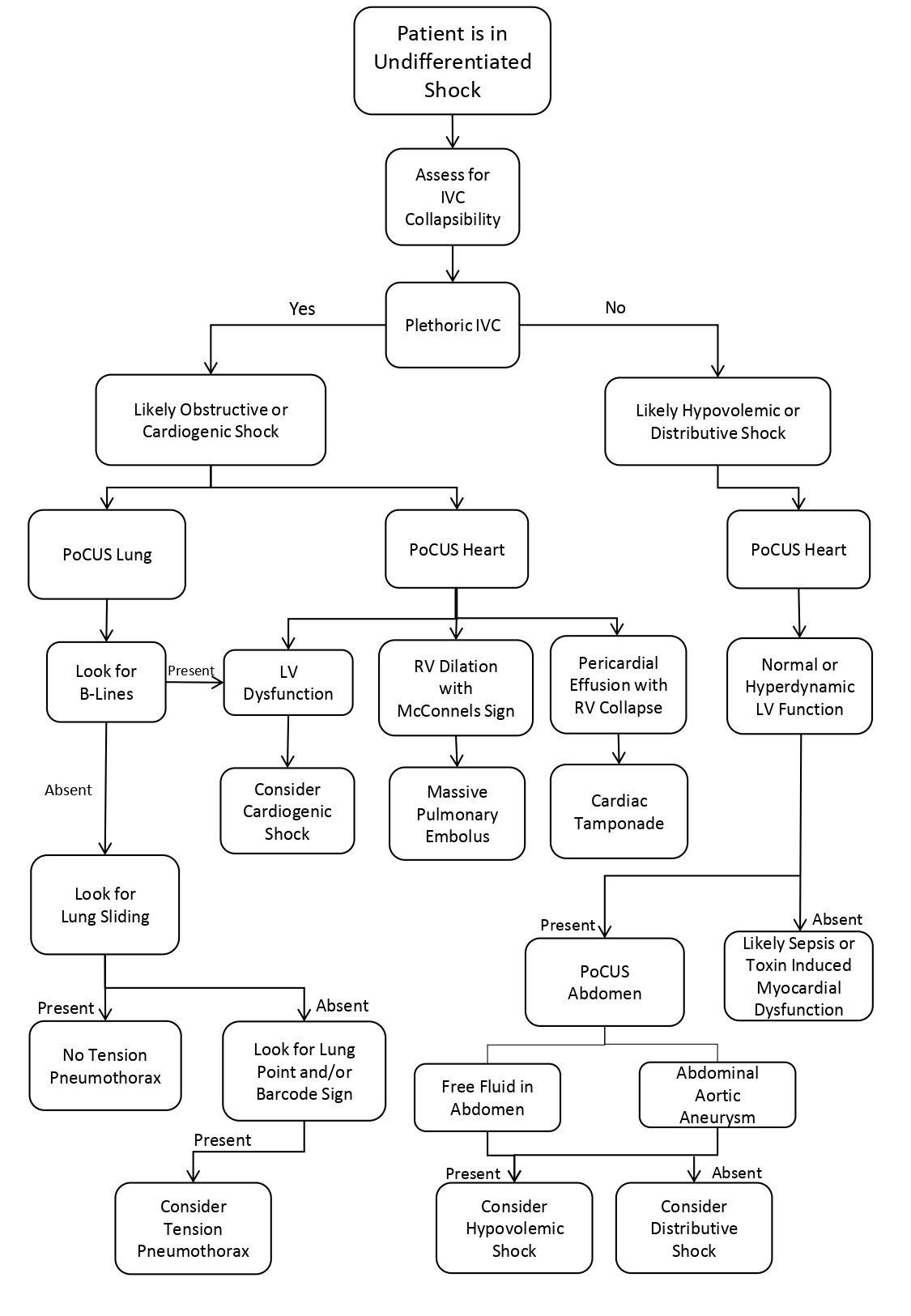


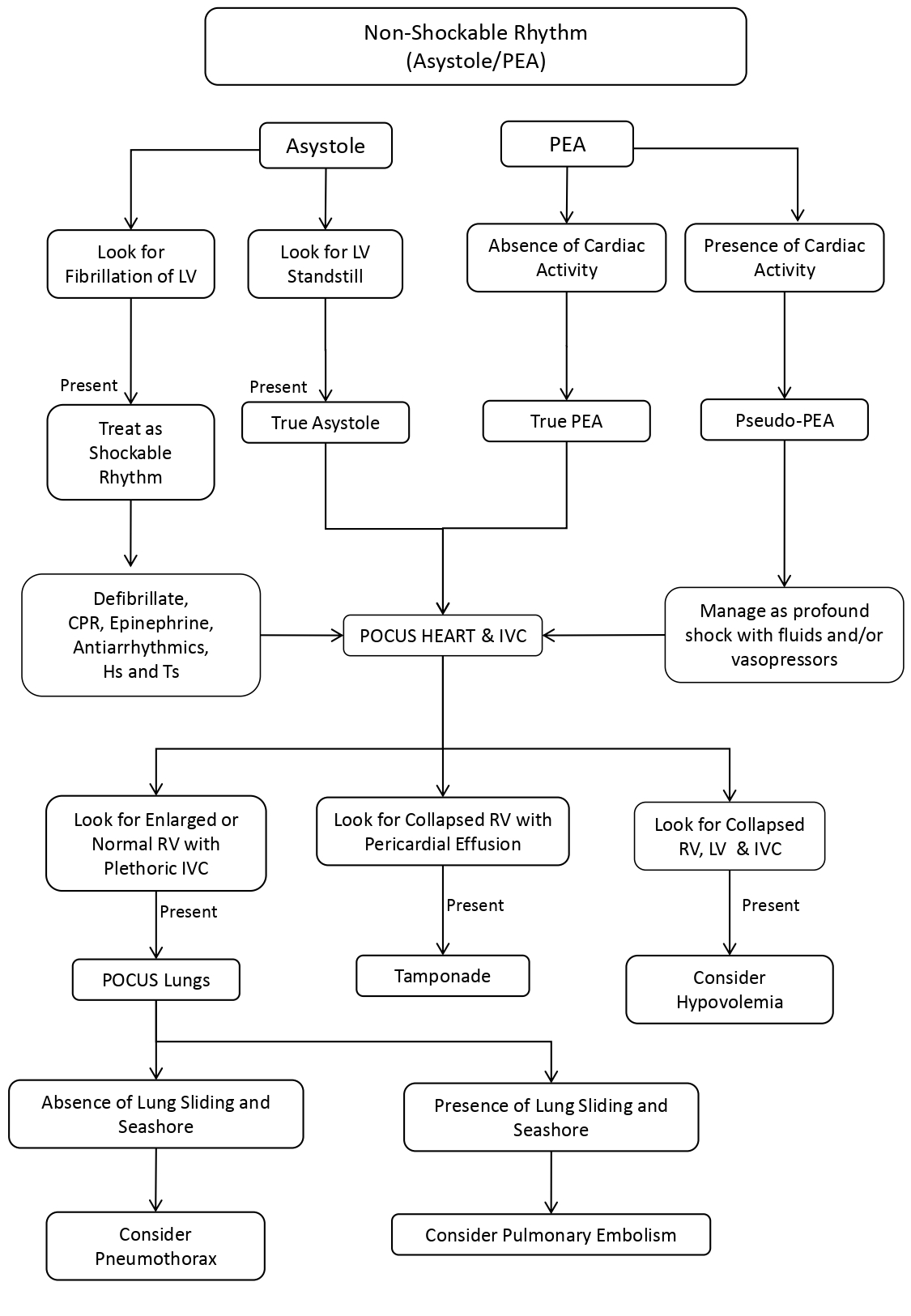

Supplement: Supplementary file 1 — Additional file 1: Supplementary Figure S1–S3. POCUS-guided algorithmic approach to manage a patient presenting with undifferentiated shock, respiratory distress, and cardiac arrest in the emergency department. [file 12245_2023_525_MOESM1_ESM.docx]
